# Supplementary material for: Beneficial impact of Ac3IV, an AVP analogue acting specifically at V1a and V1b receptors, on diabetes islet morphology and transdifferentiation of alpha- and beta-cells
Source: PLoS One. 2021 Dec 20;16(12):e0261608. doi: 10.1371/journal.pone.0261608 (PMC8687525; doi:10.1371/journal.pone.0261608)
Supplement: Attachment — Submitted filename: PLoSOne Reply.docx [file pone.0261608.s001.docx]

**Beneficial impact of Ac3IV, an AVP analogue acting specifically at V1a and V1b receptors, on diabetes islet morphology and transdifferentiation of alpha- and beta-cells by Mohan et al. PONE-D-21-20871**

Reviewer 1:

We thank the Reviewer for assessment of our manuscript, in particular his/her comments that ‘*It is new important knowledge that V1a and V1b receptor activation are related to improvement of both transdifferentiation of both alpha and beta cell*’. Our responses to the queries raised by the Reviewer are considered below.

**Specific comments**

**1.** The Reviewer comments ‘*The authors measured blood glucose and insulin levels as the effect of Ac3 IV on blood glucose level, glucose tolerance test and insulin tolerance test should be performed to evaluate glucose metabolism in more detail’*. The authors thank the Reviewer for this comment. We recognise that glucose and insulin tolerance tests are useful for evaluating overall beneficial effects of Ac3IV on metabolism. However, the primary objective of the current study was to determine the impact of Ac3IV on islet morphology, and especially transdifferentiation of alpha- and beta-cells, in our two transgenic mouse models. Thus, we have already documented positive actions of Ac3IV on glucose disposal and insulin action in HFF mice in our previously published report (please see Diabetes Obes Metab. 2021 Jun 9. doi: 10.1111/dom.14462). Also, it is technically difficult to chart increases of circulating glucose after GTT in the present STZ model due to very high starting concentrations. To highlight these matters and address the Reviewers comment, we have made the following alterations to the text:

Introduction section (Page 4, lines 13-14): ‘In this way the impact of Ac3IV on alpha- to beta-, as well as beta- to alpha-cell, transdifferentaition could be investigated, which was the primary objective of the current study’.

Discussion section (Page 14, lines 16-20): ‘Assessment of glucose and insulin tolerance would also have been useful to determine the positive impact of Ac3IV on overall metabolism, but our primary objective in the transgenic mouse models utilised for the current study was evaluation of effects on islet cell lineage. Moreover, benefits of Ac3IV on glucose homeostasis and insulin action have previously been confirmed in HFF obese mice [5]’.

**2.** The Reviewer remarks ‘*The authors used insulin and glucagon-positive cells to evaluate transdifferentiation between alpha and beta cells, but in order to obtain more detailed results, other markers such as PDX1, Nkx6.1, and Arx Expression should be assessed by staining or gene expression’*. The authors thank the Reviewer for this comment. At present we are unfortunately unable to conduct the suggested experiments. Thus, for gene expression studies in the different alpha- and beta-cell populations, namely insulin^+ve^, GFP^+ve^, insulin^+ve^, GFP^-ve^, insulin^-ve^, GFP^+ve^ , glucagon^+ve^, GFP^-ve^ cells as well as glucagon^+ve^, GFP^+ve^ cells, we would need to use cell sorting technologies that are beyond our current expertise. We are also somewhat reluctant to employ immunohistochemical staining approaches for the accurate determination of activation and levels of endogenous transcription factors. In addition, these are only predicted markers of cellular phenotype whereas we have employed a much more definitive cell lineage tracing approach using transgenic mice. However, to address the Reviewers point, we have added the following sentence to the Discussion section (Page 16, lines 12-15) that reads as follows: ‘Furthermore, although beyond the scope of the current study, consideration of levels of specific alpha- and beta-cell transcription factors such as aristaless-related homeobox (Arx), pancreatic and duodenal homeobox 1 (Pdx-1) or NK6 homeobox 1 (NKX6.1) would be useful to further assess islet cell lineage fate [7]’.

Reviewer 2:

We thank the Reviewer for their appraisal of our manuscript, in particular his/her comments that ‘*The authors suggest that Ac3IV administration improves the islet morphological changes induced by streptozotocin by Ac3IV, which is an interesting finding*’. Our responses to the queries raised by the Reviewer are considered below.

**Specific comment**

**1.** The Reviewer comments ‘*Little information on the glucagon and secondary antibodies used in this study. This is important information for multiple immunostainings. Please provide details.’*. The authors thank the Reviewer for this comment, and we are happy to comply with his/her suggestion. We have added the following additional information relating to secondary antibodies employed to the Materials and Methods section of the revised manuscript (Page 6, lines 21-23 and Page 7, lines 2-7) that reads as follows: ‘The glucagon primary antibody (PCA2/4) was raised in house in guinea-pigs immunised with porcine glucagon-carbodiimide-albumin conjugates [21], and specificity confirmed in our previous studies [22] .……..... Slides were then rinsed in PBS and incubated for 45 min at 37°C with appropriate Alexa Fluor secondary antibodies (1:400; Invitrogen, Alexa Fluor 488 for green or 594 for red, Invitrogen). The following secondary antibodies were employed, as appropriate, goat anti-mouse Alexa Fluor 488, goat anti-mouse Alexa Fluor 594, goat anti-guinea pig Alexa Fluor 488, goat anti-guinea pig Alexa Fluor 594, donkey anti-goat 488 and goat anti-rabbit Alexa Fluor 488’. The reference section has been updated accordingly.

**2.** The Reviewer remarks ‘*. In Fig.3D and Fig.7C/D, the degree of decrease in β-cell area by STZ administration and the degree of recovery by Ac3IV administration are quite different. Can this be explained by the difference in the mice used?’*. The authors thank the Reviewer for this interesting comment and insightful suggestion. Thus, we have reported previously a more severe STZ-induced diabetic phenotype in *Ins1*^CRE/+^;*Rosa26-eYFP* when compared *Glu^CreERT2^*;*Rosa26-eYFP* mice (Lafferty et al. *Front Endocrinol (Lausanne)*. 2021; 12:633625). This was suggested to occur because of variations in STZ susceptibility between the strains of mice. The observation fully accords with greater reductions in islet and beta-cell areas observed in the current study in *Ins1*^CRE/+^;*Rosa26-eYFP* (Figure 3B,D) when compared to *Glu^CreERT2^*;*Rosa26-eYFP* STZ-diabetic mice (Figure 7A-D). To follow up on the Reviewer’s comment, we have added the following text to the Discussion (Page 16, lines 8-12), that reads as follows: ‘However, the established detrimental effects of STZ on islet architecture were apparent to a greater extent in *Ins1*^CRE/+^;*Rosa26-eYFP* mice, in agreement with recent observations of a more severe STZ-induced phenotype in these mice compared with *Glu^CreERT2^*;*Rosa26-eYFP* diabetic mice [15]. This difference was suggested to be linked to variations in STZ susceptibility between the two strains of mice’.

**3.** The Reviewer comments ‘*In Fig. 4C, the percentage of insulin+, GFP- cells increased to about 40% in Ac3IV administrated group, but in the representative image of Fig. 4A, the percentage of insulin+, GFP- cells does not seem to be 40%. Also, the sum of the cells in Fig.4C and D, STZ treated group exceeds 100%. What does the vertical axis represent? Moreover, insulin+ and GFP+ β-cells should account for a large percentage of the cells, this does not appear to be the case in the graphs of Fig. 4C-E. The authors should describe the analysis method and what the vertical axis represents in detail*’. The authors thank the Reviewer for this comment and the opportunity to clarify these matters. We agree that the image displayed in Figure 4A is not a perfect representation of approximately 40% insulin^+ve^, GFP^-ve^ cells in Ac3IV treated mice. We have now replaced this image with a more representative picture of the islets. With regards to the sum of the cells in Figure 4C&D, in panel C, the population of cells that express insulin only are being assessed, whereas as in panel D we are quantifying a different population of cells that only express GFP. As such, these are distinct populations of cells that we would not expect to add up to 100% in total for each treatment group. To help simplify this matter, we have now added small titles above each figure panel to make these graphs easier to interpret. Panel C is now labelled ‘Insulin-expressing non-beta cells’, panel D ‘Insulin-deficient beta-cells’ and panel E ‘Glucagon expressing beta-cells’. Finally, we fully agree that insulin^+ve^, GFP^+ve^ cells should account for a large percentage of islet cells, and this is in fact the case. To note, we have not specifically reported on numbers of insulin^+ve^, GFP^+ve^ cells in Figure 4, as this is simply the inverse of insulin^-ve^, GFP^+ve^ (Figure 4D) cells. For example, in panel D the saline treatment group expresses around 20% insulin^-ve^, GFP^+ve^ beta-cells, which implies that the remaining 80% of GFP^+ve^ would be insulin^+ve^. We are confident that the new titles above each of the panels in Figure 4 will help with overall interpretation of these data. The authors thank the Reviewer for the chance to improve the quality and clarity of our figures.

**4.** The Reviewer notes ‘*The authors do not discuss the mechanisms by which Ac3IV treatment increases alpha cell apoptosis and decreases proliferation. The authors should discuss it’*. The authors thank the Reviewer for this comment and are happy to comply with his/her suggestion. In general, activation of V1a and V1b receptors is linked to cell pro-survival effects, both in normal islet cells [Biochimie. 2019; 158:191-198] as well as in epithelial cells, glomerular mesangial cells and vascular smooth muscle [Front Med (Lausanne). 2015; 24:19]. This is suggested to be linked to V1a and V1b Gq receptor coupling and activation of the phospholipase C (PLC) pathway. In disease states such as diabetes, combined V1a and V1b receptor activation has been shown to result in decreased proliferation of both pancreatic alpha- and beta-cells [Diabetes Obes Metab. 2021 Jun 9. doi: 10.1111/dom.14462]. In keeping with this, the impact of Ac3IV to increase apoptosis and decrease proliferation of alpha-cells, most likely relates to the disease phenotype induced by STZ administration in the current setting. As such, STZ administration is well established to reduce islet beta-cell mass and dramatically increase alpha-cell mass. Therefore, the impact of Ac3IV on alpha-cells most likely reflects promotion of a more normal islet architecture. To better highlight the matter, we have added the following sentence to the Discussion section of the revised text, that reads (Page 13, lines 14-18): ‘More notably, the decreases of alpha-cell area and pancreatic glucagon were accompanied by enhanced alpha-cell apoptosis and tendency for decreased alpha-cell proliferation. This is interesting given that V1a and V1b receptor activation is generally associated with pro-survival effects [2], linked to Gq receptor coupling and activation of the phospholipase C (PLC) pathway. Effects of Ac3IV in the current setting may simply represent the capacity for Ac3IV to reverse the detrimental effects of STZ on pancreatic islet architecture. Indeed, similar effects of Ac3IV to decrease alpha-cell growth have been demonstrated in HFF mice [5]’.

**5.** The Reviewer comments ‘*The values of the vertical axis in Fig.7G and H are much larger than those in Fig.7A and B. I wonder why the area of large islets (>25000 μm2) is larger than the islet area shown in Fig.7A and B. The authors should explain in detail how to analyze the graph and what the vertical axis represents’*. We thank the Reviewer for the opportunity to explain this point. He/she is correct that our original graph axes labelling, and subsequent presentation of data, could lead to some confusion within Figure 7. We apologise for this, but are grateful that we can now correct the matter. In the original version of Figure 7, panels G&H, islet size distribution data was simply displaying the average size of small (< 10,000 µm^2^), medium (10,000 – 25,000 µm^2^) and larger (> 25,000 µm^2^) sized islets, with no indication of the percentage of total islets within each islet size group. We have now converted these data to depict a percentage of total islets analysed. We are confident that this change has addressed the Reviewers concern.

**6.** The Reviewer further comments ‘*Different results are shown between the head and tail of the pancreas regarding the effects of AC3IV, such as glucagon content and islet size distribution. Is there any relationship between the distribution of receptors or other factors that could be the reason for these results? Please discuss it’*. We thank the Reviewer for this comment and the opportunity to further discuss this matter. As noted in the original manuscript, pancreatic islets are more concentrated and richer in alpha-cells in the tail, as opposed to the head, of the pancreas. To the best of our knowledge, there is no definitive literature to suggest differences in receptors or other factors between the head and tail portions of the pancreas. However, in the context of the current study and islet cell lineage tracing, it may be of interest to note that the head and the tail of the pancreas have different developmental origins. Thus, the head of the pancreas is formed from the dorsal and ventral pancreatic bud, and the tail from the ventral pancreatic bud only. In addition, previous studies in rodents have shown that islets originating from the dorsal pancreatic bud have greater capacity to secrete and synthesise insulin than islets of ventral bud origin. We have therefore added the following sentences to the Discussion section of the revised manuscript in relation to this information, that read as follows (Page 15, lines 7-12): ‘Furthermore, in terms of effects of Ac3IV on islet cell lineage it may be of interest to note that the head and the tail of the pancreas have different developmental origins. As such, the head of the pancreas is formed from the dorsal and ventral pancreatic bud, whereas the tail is derived from the ventral pancreatic bud [30]. Moreover, there is a suggestion that islets from the head of the pancreas contain more glucagon and somatostatin and have a greater insulin secretory capacity than those originating solely from the ventral bud region [31]’. The reference section has been updated accordingly.
