# Supplementary material for: Beneficial impact of Ac3IV, an AVP analogue acting specifically at V1a and V1b receptors, on diabetes islet morphology and transdifferentiation of alpha- and beta-cells
Source: PLoS One. 2021 Dec 20;16(12):e0261608. doi: 10.1371/journal.pone.0261608 (PMC8687525; doi:10.1371/journal.pone.0261608)
Supplement: Attachment — Submitted filename: PLoSOne Reply_2.docx [file pone.0261608.s002.docx]

**Beneficial impact of Ac3IV, an AVP analogue acting specifically at V1a and V1b receptors, on diabetes islet morphology and transdifferentiation of alpha- and beta-cells by Mohan et al. PONE-D-21-20871R1**

Reviewer 1:

We are pleased to note Reviewer 1 has no further comments and is happy with our revised manuscript.

Reviewer 2:

We thank the Reviewer for their reappraisal of our revised manuscript, and particularly the following comment: ‘*Thank you to the authors for their careful consideration and answers to the previous comments. The manuscript has greatly improved*’. Our response to the one remaining query raised by the Reviewer is considered below.

**Specific comment**

**1.** The Reviewer notes ‘*I only have one but an important comment. Previous Comment No.5: Fig. 7G was made clearer by the authors' revision. However, it seems that the intention of my question was not well understood by the authors. Regarding Fig.7 G and H, the authors classified the islet into small (< 10,000 μm2), medium (10,000-25,000 μm2) and large (> 25,000 μm2) islets. However, in Fig.7A/B and Fig.3B, the graphs show that the area of islets and the maximum value is about 10,000 μm^2^. It is questionable that these areas are much smaller than the average area of a single small size islet. What does the islet area in Fig.7A/B and Fig.3B represent? If it represents the area of islets per unit area of pancreas, please describe it that readers can understand it’*. The authors agree that on first look there may appear to be a small discrepancy between the islet areas depicted within panels A&B of Figure 7, versus panels G&H. We are pleased for the opportunity to clarify this matter and remove any ambiguity. By way of explanation, within the group of islets classified as small in Figure 7G&H, that is < 10,000 μm^2^ in area, a large proportion of these islets are actually less than 2,000 μm^2^. This accounts for the seemingly low overall average islet and beta cell areas depicted in Figure 7A&B. To make this more apparent, we have added an extra classification of ‘very small islets’, of < 2,000 μm^2^ in area, to our data analyses within Figure 7G&H. We are confident that this change has now addressed the Reviewers concern. To fully highlight this change we have made the following alterations to the Results section of the manuscript (Page 11, line 23 and Page 12, lines 1-6), that reads: ‘The number of very small (<2,000 µm^2^), small (2,000 - 10,000 µm^2^) and medium (10,000 - 25,000 µm^2^) sized islets was similar across all groups of mice in the head of the pancreas, but larger (>25,000 µm^2^) sized islets were only detectable in Ac3IV treated mice (Fig. 7G). Islet size distribution was not noticeably different between all the various groups in pancreatic tail, but Ac3IV treated mice did appear to have reduced numbers of very small (<2,000 µm^2^) islets, with larger sized islets being more prevalent (Fig. 7H)’. No further alterations to the text are required as overall interpretation of these data has not changed.
